# Aptamer-linked immobilized sorbent assay for detection of VP1 of foot and mouth disease virus serotype O

**DOI:** 10.1038/s41598-025-34793-8

**Published:** 2026-02-03

**Authors:** Irwin A. Quintela, Raymondo Lopez-Magaña, Anya Hwang, Tyler Vasse, Vivian C.H. Wu

**Affiliations:** Produce Safety and Microbiology Research Unit, US Department of Agriculture, Agricultural Research Service, Western Regional Research Center, Albany, 94710 CA USA

**Keywords:** Foot and mouth disease (FMD), Aptamers, Senecavirus A (SVA), ALISA, VP1, Biochemistry, Biological techniques, Biotechnology, Diseases, Microbiology

## Abstract

Foot and Mouth Disease (FMD) is one of the most devastating animal diseases. Infected animals, such as swine, develop vesicles, often leading to morbidity or depopulation of exposed herds. Current FMD diagnosis relies on serologic and immuno-based methods, which commonly encounter cross-reactivity and cost issues. Therefore, a simple and portable diagnostic tool would greatly aid its detection efforts. This study generated a novel aptamer sequence targeting viral protein 1 (VP1) of serotype O FMD virus (FMDV) by Systematic Evolution of Ligands by Exponential Enrichment (SELEX). The generated FMDV VP1-specific aptamer sequence was characterized and incorporated into an Aptamer-linked Immobilized Sorbent Assay (ALISA)-based microplate platform to detect FMDV VP1. Results showed that the aptamer, FMDV Apt, had an estimated dissociation constant (*K*_d_) of 14 ± 8.6 nM, showing strong affinity for VP1 of FMDV. A concentration-dependent linear relationship (R^2^ = 0.9354) within a concentration range of 0.50 ng/mL – 5.0 ng/mL of FMDV VP1 protein was observed. The limit of detection (LOD) was determined to be 1.3 ng/mL, and the limit of quantification (LOQ) was approximately 4 ng/mL. Moreover, the FMD ALISA did not exhibit cross-reactivity when tested with high concentrations (500 ng/mL – 2,000 ng/mL) of non-targets, indicating its robustness and potential application in other detection platforms. The newly developed screening tool would facilitate efficient and inexpensive monitoring of potential biohazard risks posed by FMDV.

**Supplementary Information:**

The online version contains supplementary material available at 10.1038/s41598-025-34793-8.

## Introduction

Foot and Mouth Disease (FMD) is one of the most feared livestock diseases due to its high infectivity rate and severely damaging effects on animals^1,2^. Both wild and domestic cloven-hoofed animals, including sheep, goats, cattle, and pigs, are prone to FMD Virus (FMDV) infection, which can eventually lead to extensive meat and dairy production losses^1,3^. FMD is a transboundary livestock disease with the potential to spread to many FMD-free countries, such as North America and Europe^4,5^. Given the devastating impact of FMD outbreaks, early and accurate detection of FMDV is critical for effective disease control and prevention. As a positive-sense and single-stranded RNA virus, FMDV forms an icosahedral protein capsid which consists of four major viral proteins (VP1 – VP4)^6^. Viral protein 1 (VP1) is a surface-exposed protein and the major antigen of FMDV capsid protein^6–8^. Specifically, VP1 allows attachment to host cells through integrin receptors^9^. Among the seven FMDV serotypes (A, C, O, Asia 1, SAT1 – SAT3) and subtypes, serotypes O and A have the broadest spatiotemporal distribution^7–9^. These features make VP1 a target candidate for the development of diagnostics. FMDV-infected animals develop lesions on their oral cavities, tongues, muzzle, teats, and coronary bands^10^. However, these vesicular lesions are unreliable clinical symptoms for diagnosis purposes due to shared similarities with other viral lesions, including lesions resulting from vesicular diseases in swine caused by Senecavirus A (SVA) and vesicular stomatitis in cattle caused by vesiculovirus^5,10^.

Currently, laboratory-based diagnostics include serologic methods for FMDV infection-associated antigen detection, while enzyme-linked immunosorbent assay (ELISA) and complement fixation are employed to test for FMDV antigen^10^. Additionally, ELISA can differentiate antibody titers collected from vaccinated and infected herds^10^. However, ELISAs can elicit cross-reactivity, require specialized equipment and training, and are costly^10–12^. Thus, since FMD is highly contagious for cloven-hoofed animals, fast and early detection of FMDV is critical for its control and mitigation. These drawbacks are particularly problematic in resource-limited regions, where rapid and low-cost detection tools are urgently needed.

In areas where both staff and resources are limited, alternative methods and technologies with inexpensive, sensitive, and short turnaround times are urgently needed. Aptamers, which are short single-stranded oligonucleotides, can act as a non-immunological-based option for detection^13^. Compared to antibodies, aptamers exhibit target flexibility, allowing them to recognize viruses, whole cells, and small molecules with high affinity and specificity^11,12,14^. Aptamers offer other advantages over antibodies, such as a low degree of immunogenicity, higher stability, longer shelf life, cell-free evolution, no batch-to-batch variation, low production cost, and proven sustainability due to their in vitro developmental procedures^15^.

In this study, an aptamer sequence targeting VP1 of serotype O, one of the most prevalent FMDV serotypes with distinct lineage and focus of this study, was generated by Systematic Evolution of Ligands by Exponential Enrichment (SELEX). The generated VP1-specific aptamer sequence was characterized and incorporated into an Aptamer-Linked Immobilized Sorbent Assay (ALISA) based microplate platform to detect FMDV. This platform allows high sensitivity and rapid testing at low cost, distinguishing it from traditional diagnostics. The newly developed screening tool would facilitate efficient and inexpensive monitoring of potential biohazard risks posed by FMDV. This study contributes to improving FMD surveillance capabilities, particularly in regions with limited laboratory resources, and supports timely intervention to mitigate the spread of FMDV.

## Results

### SELEX enrichment and validation

**Fig. 1:**
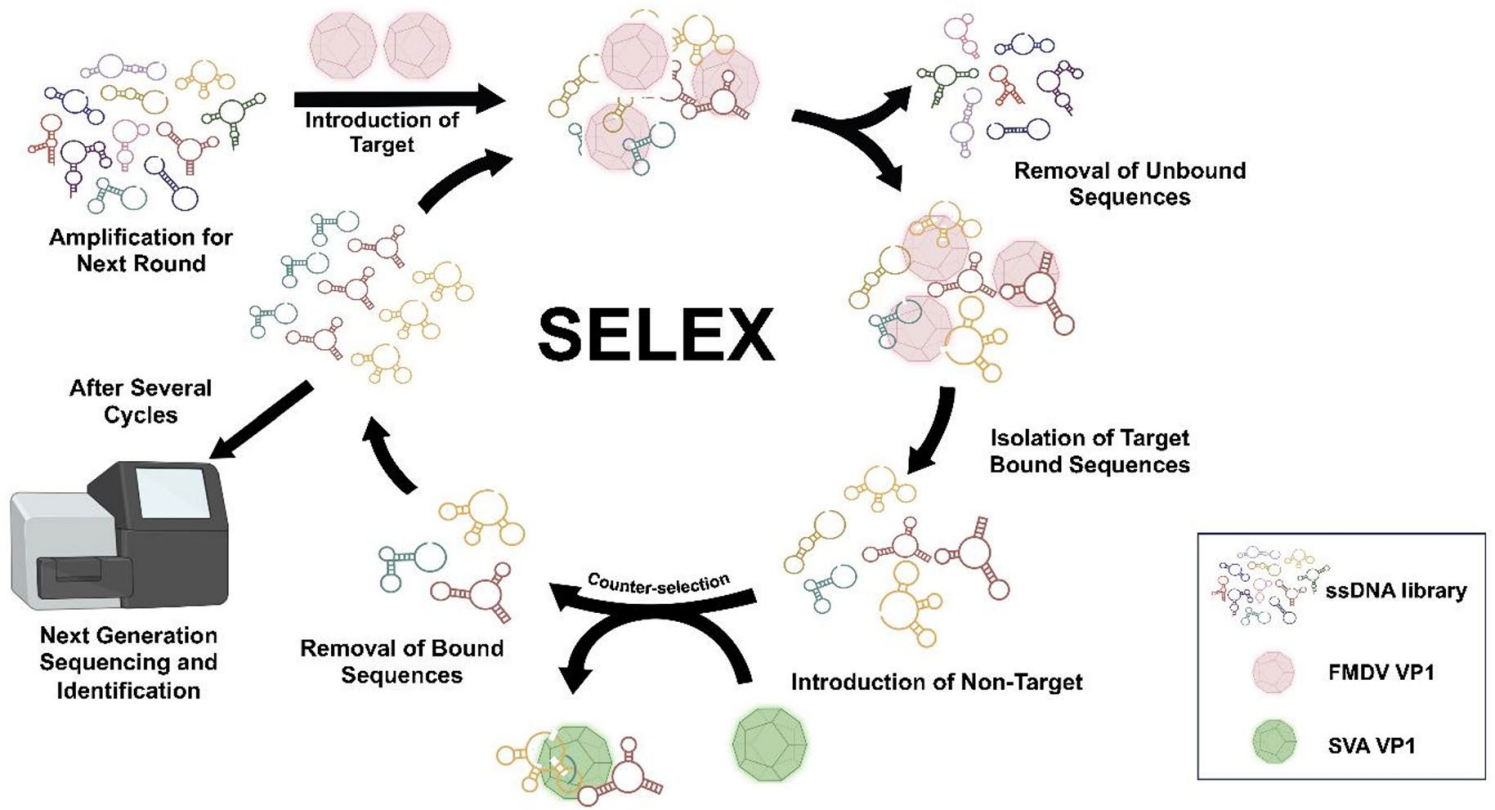
SELEX workflow used in the study with minor modifications^34^. FMDV VP1 and SVA VP1 were used as the protein baits for positive and negative selections, respectively. Adapted from Quintela et al., 2025, *Elucidating the molecular docking and binding dynamics of aptamers with spike proteins across SARS-CoV-2 variants of concern*,* Frontiers in Microbiology*, 2025, 10.3389/fmicb.2025.1503890, under the terms of the Creative Commons Attribution License (CC BY 4.0).

In this study, a two-step PCR procedure was conducted in each round of SELEX. The SELEX workflow is illustrated in Fig. 1. Initially, a preparatory PCR was performed to determine the optimal cycle number for the subsequent amplification PCR, during which the sub-library was generated. The optimum cycle number was defined as the number of PCR cycles that yielded the highest amount of aptamer fragments while minimizing the presence of PCR byproducts. The optimal PCR cycles were determined to be with 18–20 cycles, yielding the highest target ssDNA with minimal byproducts, as shown in Supplementary Figure S1. After eight selection cycles, the generated ssDNA pools were processed and sequenced. The most prevalent sequence (81-nt), named FMDV Apt, (5’ ATC CAG AGT GAC GCA GCA AGA GAC CTG CAA GGC AAG CGA TTT AAG TGG CAC CCC CAG GGA CCA TGG ACA CGG TGG CTT AGT – 3’) was chosen for further characterization. These results suggest that FMDV Apt binds VP1. Generation of aptamers by SELEX identified FMDV Apt as a candidate for use as a capture molecule against VP1, providing support for structural and docking analysis.

### Structural Characterization and Molecular Docking

**Fig. 2:**
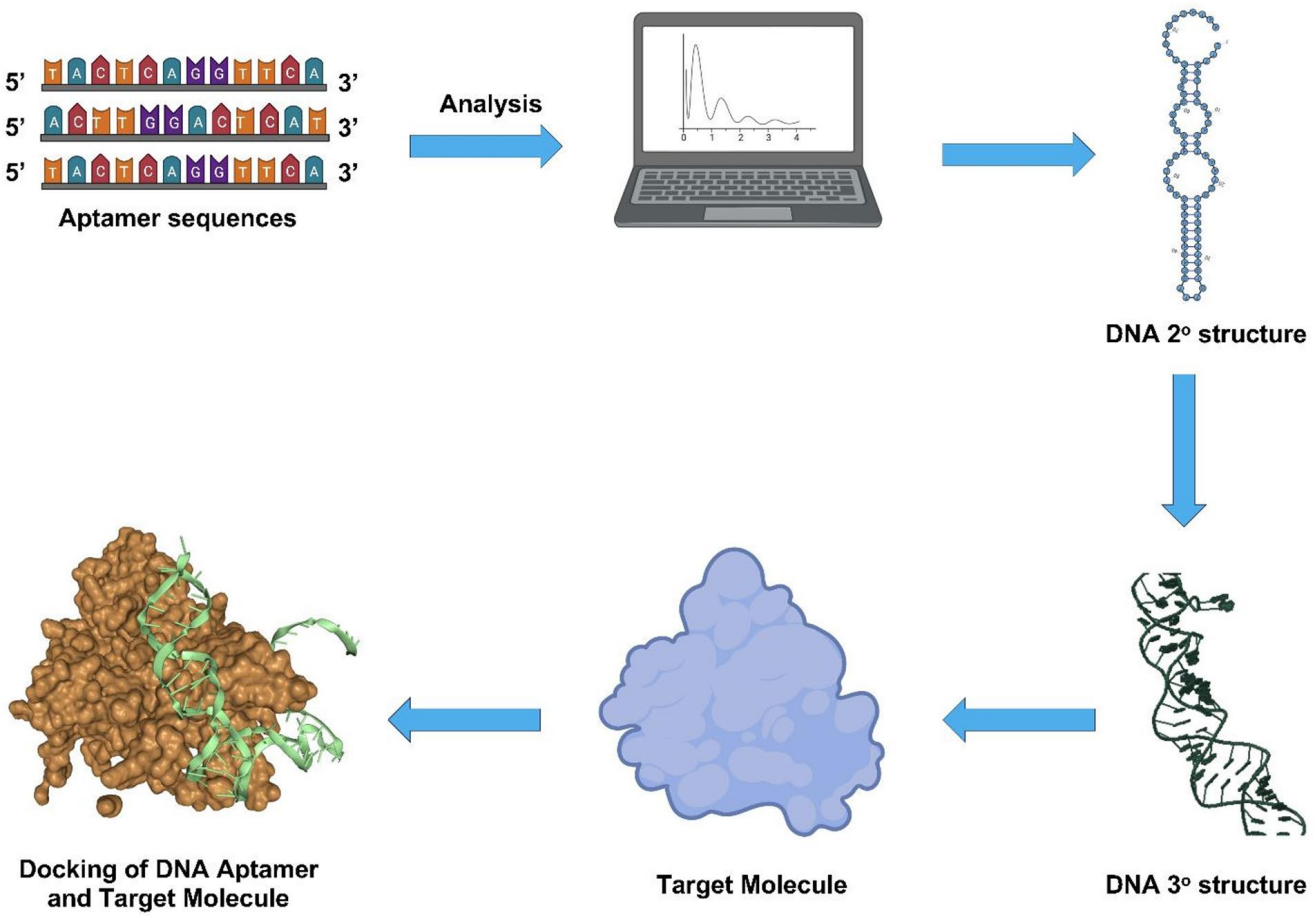
Workflow of aptamer sequence characterization. Processing workflow from NGS data, predicting secondary and tertiary structures, and molecular docking simulation. Adapted from Quintela et al., 2025, *Elucidating the molecular docking and binding dynamics of aptamers with spike proteins across SARS-CoV-2 variants of concern*,* Frontiers in Microbiology*, 2025, 10.3389/fmicb.2025.1503890, under the terms of the Creative Commons Attribution License (CC BY 4.0).

**Fig. 3:**
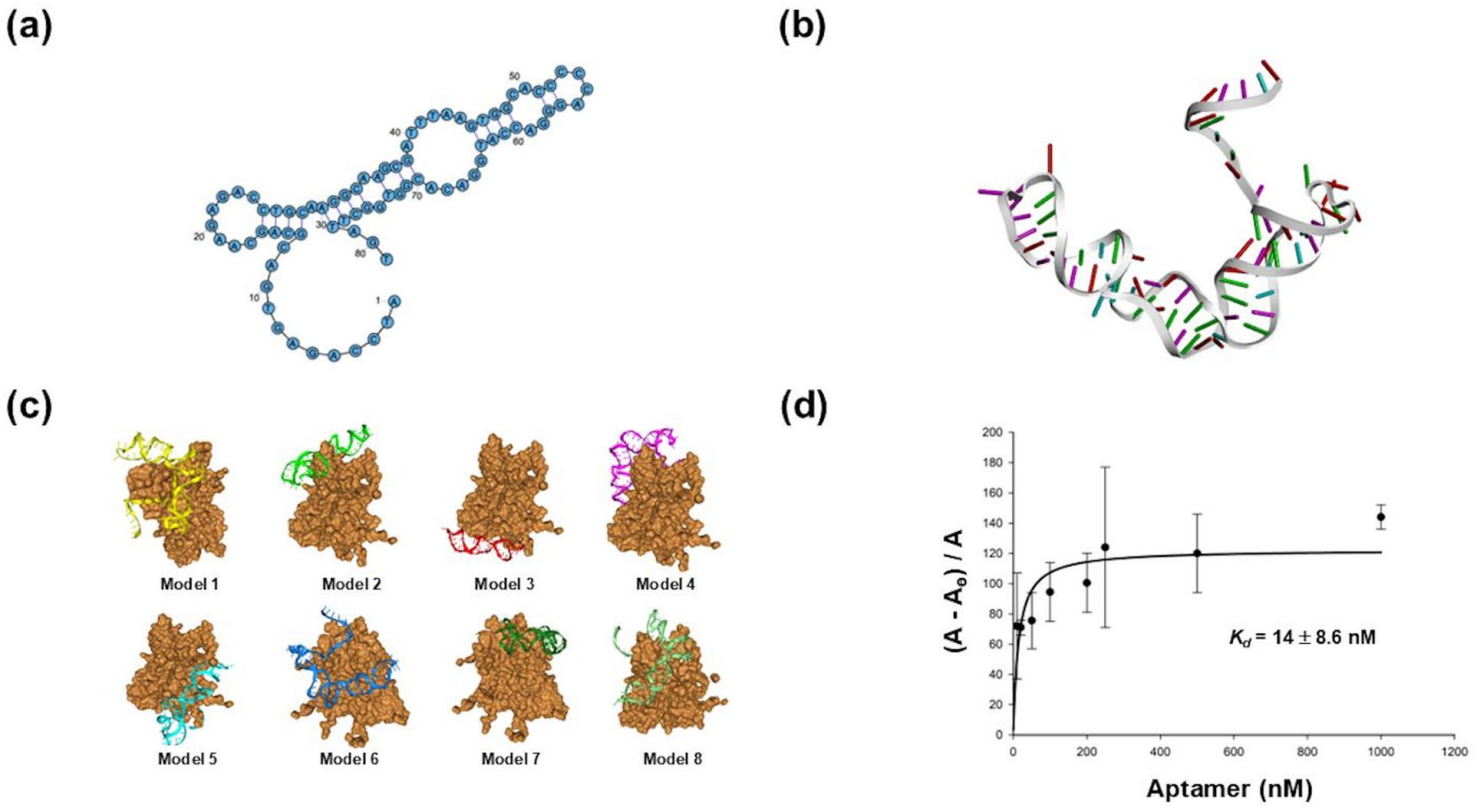
Structures and selection of aptamers from a random library. (**a**) Predicted secondary structure of FMDV Apt (**b)** Predicted tertiary structure of FMDV Apt (**c**) Top eight models of molecular docking simulation as predicted by HDOCK^19^ (**d**) Binding affinity curve and *K*_d_ of FMDV Apt. Binding affinity curve is representative of 3 biological replicates (*n* = 3) with error bars indicating the mean ± S.D.

To begin our characterization of FMDV Apt, we analyzed its folding properties and docking potential. Accordingly, we subjected FMDV Apt to secondary structure analysis using RNAsoft CombFold^16^ and RiboSketch^17^ to generate and visualize predicted structures. This approach, shown in Fig. 2, identified various predicted secondary structure models, which we ranked based on the lowest Gibbs free energy. The model with the lowest Gibbs free energy of ∆G = -3.95 kcal/mol (Fig. 3(a)) was selected. This score, when compared to the other models, suggests this predicted secondary structure displays the greatest thermodynamic stability. This result suggests that this folding motif is the most stable secondary structure.

We then predicted the tertiary structure using Discovery Studio Visualizer (Biovia) and CHARM GUI^18^, followed by molecular docking analysis by HDOCK^19^, as previously reported. Structural motifs in higher-order nucleotide structures, including hairpin loops, pseudoknots, and stems, serve as binding elements for molecules like proteins^15,20^. The tertiary structure of FMDV Apt includes a stem and hairpin loops (Fig. 3(b)), consistent with the secondary structure. These motifs serve as potential structural binding elements for VP1 where FMDV Apt may bind. Subsequently, this structure was used for molecular docking analysis^19^ of FMDV Apt to the FMDV VP1 protein. Docking scores, confidence scores, and ligand root-mean-square deviation (RMSD)^21,22^ are presented on Supplementary Table S2. The docking scores (−301.3 to −263.07) were calculated by an iterative scoring feature of HDOCK that facilitates ranking of potential binding models based on the negative score values. All these binding models display a confidence score > 0.90, suggesting a high likelihood of FMDV Apt binding to VP1. For the ligand RMSD, the values ranged from 473.65 Å − 518.16 Å. These values provide a meaningful assessment of the alignment between the reference and predicted structures. Lower ligand RMSD values indicate closeness of the docked models to the reference structures, while higher values suggest greater structural deviation. The top model revealed binding at VP1’s receptor binding domain via hydrogen bonding and π-π stacking (Fig. 3(c)). In addition, molecular docking of FMDV Apt to VP1 revealed that this aptamer indeed forms structural motifs, including hairpin loops, stems, and pseudoknots (Fig. 3(c)). Hairpin loops provide structural flexibility for interaction with VP1’s epitopes. Overall, the results suggest that the aptamers’ structural motifs facilitate binding to VP1 through a combination of electrostatic interactions, hydrogen bonding, π-π stacking, hydrophobic interactions, and Van der Waals forces^15,20^. These findings suggest that FMDV Apt binds the FMDV VP1 protein. Structural analysis confirmed the presence of thermodynamically stable motifs, laying the foundation for specific VP1 binding.

### Binding affinity determination

Because our structural and docking analysis suggests an interaction between FMDV Apt and the VP1 protein of FMDV (Figs. 3(a-c)), we next assessed their binding potential by magnetic bead-based isocratic elution^23^. The fluorescence intensity was plotted as a function of FMDV Apt concentration (Fig. 3(d)) to calculate the dissociation constant (*K*_d_) of FMDV Apt to FMDV VP1. Using Eq. (1), FMDV Apt was found to have a *K*_d_ of 14 ± 8.6 nM. Although the standard deviation (S.D.) is approximately 60% from the calculated *K*_d_, this is typical for this type of affinity estimation^24^. This large *K*_d_ S.D. may be attributed to technical variability from magnetic bead elution efficiency, supported by consistent binding trends across replicates. Moreover, the calculated *K*_d_ falls within the range of other characterized FMDV aptamers, albeit these target different proteins^25,26^. This result suggests that FMDV Apt binds the VP1 protein of FMDV. The binding of FMDV Apt to VP1 with high affinity provides support for the use of FMDV Apt in a detection platform.

### ALISA sensitivity and specificity

**Fig. 4:**
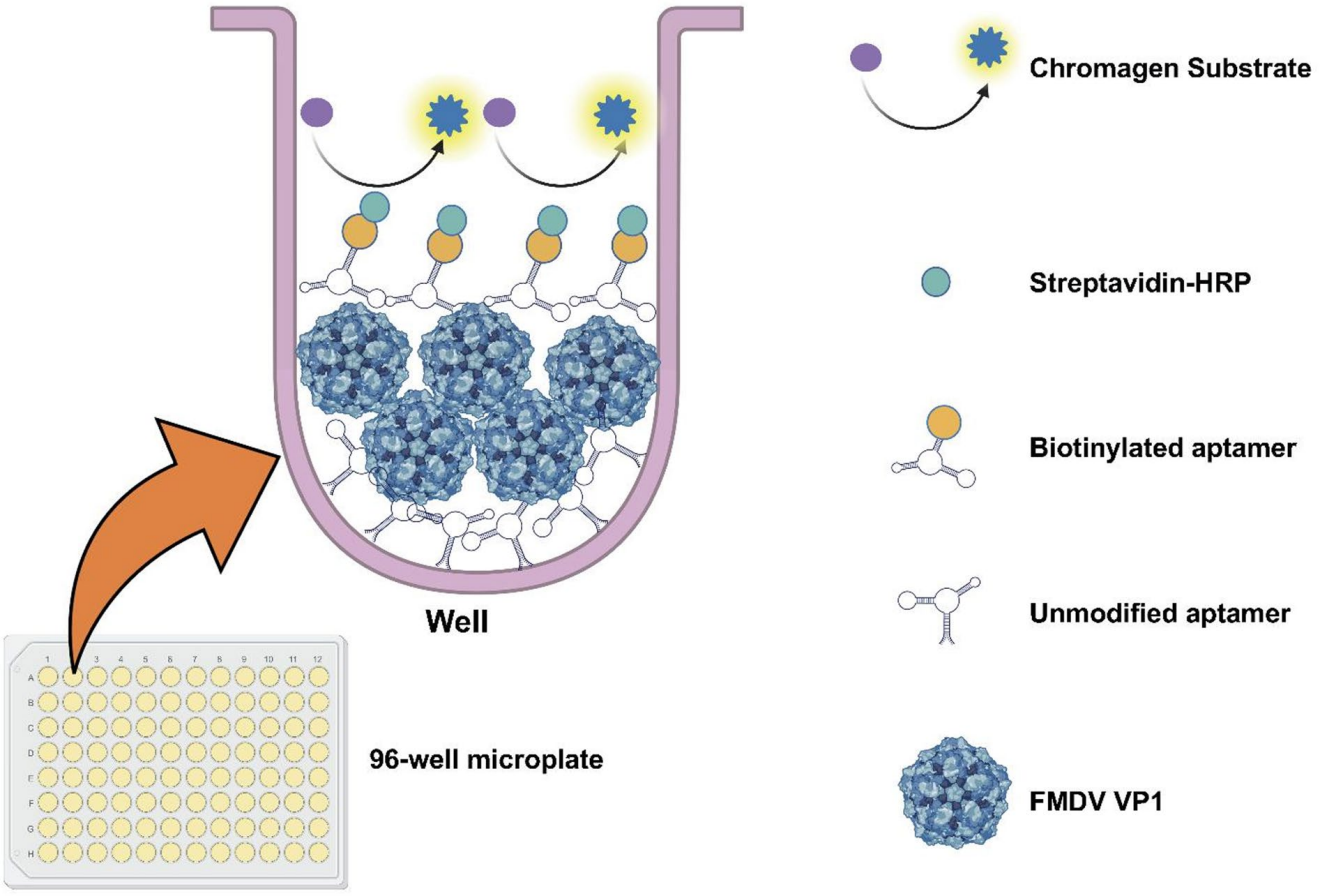
ALISA model and principles. Representation of ALISA principles showing its components and binding processes^23^.

**Fig. 5:**
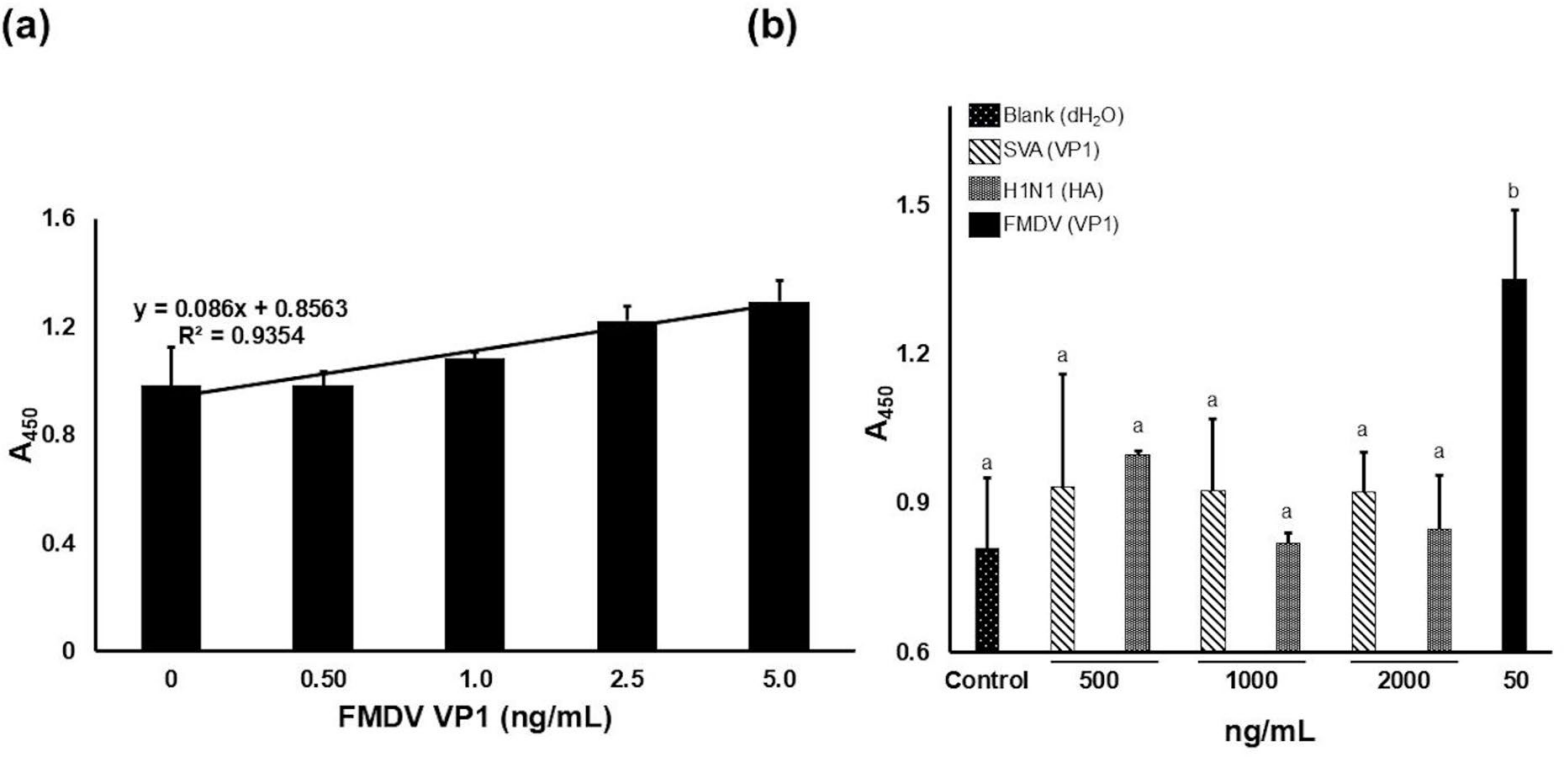
Sensitivity and specificity of FMDV ALISA. **(a**) Absorbance response graph of FMDV Apt with various concentrations of FMDV VP1. Linear regression analysis using Excel was performed to generate the calibration curve (**b**) Signals generated at A_450 nm_ of target (FMDV VP1), non-target samples (SVA VP1 and H1N1 HA), and no target negative control. A_450 nm_ is calculated from 3 biological replicates with error bars indicating the S.D. Graphs are representative of 3 biological replicates (*n* = 3). a and b, *p* < 0.05.

To further characterize FMDV Apt-FMDV VP1 binding, we developed an ALISA detection platform (Fig. 4) to evaluate the sensitivity and specificity. Linear regression analysis of absorbance signals of FMDV Apt at 450 nm (A_450 nm_) shows a linear, concentration-dependent relationship within the range 0.5 ng/mL – 5 ng/mL of FMDV VP1, (y = 0.086x + 0.8563, R² = 0.9354) (Fig. 5(a)). The calculated LOD and LOQ were determined to be 1.3 ng/mL and 4 ng/mL, respectively (Supplementary Table S3(a – d)). The specificity of the FMDV aptamer-based ALISA was assessed using recombinant proteins from a related *Picornaviridae* virus (SVA VP1) and an unrelated *Orthomyxoviridae* virus (H1N1 hemagglutinin (HA)), as both cause similar clinical symptoms to FMDV (Fig. 5(b)). At 50 ng/mL concentration of FMDV VP1, the average A_450 nm_ was 1.4 ± 0.14. In contrast, even with high concentrations, the average A_450 nm_ of non-target samples remained significantly lower: for SVA VP1 500 ng/mL = 0.93 ± 0.22, 1,00 ng/mL = 0.92 ± 0.14, and 2,00 ng/mL = 0.92 ± 0.08; for H1N1 HA 500 ng/mL = 0.99 ± 0.007, 1,000 ng/mL = 0.81 ± 0.02, and 2,000 ng/mL = 0.84 ± 0.10 (Fig. 5(b)). These findings suggest that the ALISA platform specifically detects the VP1 protein of FMDV. One-way ANOVA analysis showed a significant increase in A_450 nm_ when FMDV VP1 was the target protein compared to the non-targets. Notably, increasing the non-target protein concentrations up to 2,000 ng/mL did not produce substantial responses within the FMDV VP1 range.

Altogether, FMDV Apt shows a strong binding affinity and absence of cross-reactivity by preferentially recognizing FMDV VP1 at low concentrations over non-target proteins.

## Discussion

A common source of failure in SELEX is the generation of PCR byproducts, which result in the formation of DNA amplicons containing extraneous base pairs^27^. We chose to lower the PCR cycle number and include an additional amplification PCR step, as previously reported^28^, to limit byproduct formation and generate sufficient amounts of the desired aptamer. Additionally, TBE-Urea gel separation was performed to remove nonspecific DNA and yield the desired products for subsequent rounds of SELEX. After observing that there was no additional improvement in the binding capacity between ssDNA pools from 2 successive rounds, the selection was completed at round 8.

Various strategies were employed during aptamer generation to select aptamers with the greatest affinity for VP1 of FMDV. First, an aptamer-to-target ratio of 50:1 was used as previously reported^29,30^. However, this ratio gradually decreased in subsequent rounds of incubation^28^. Secondly, the incubation time decreased from 1 h in the first round of SELEX to 10 min in the eighth round of SELEX. Furthermore, the binding buffer was supplemented with MgCl_2_, as it is reported to stabilize aptamer secondary structure and enhance binding^29^. Another important step in the selection design was the incorporation of a negative selection process. Negative selection is the selection against non-target components and materials with structures closely identical to the FMDV VP1 protein in complex matrices. Altogether, the modifications made throughout SELEX created more stringent conditions, ensured the high binding of aptamers to the protein target, and ensured that the identified aptamer was specific to FMDV VP1.

Sequence features about aptamers facilitate the clustering of aptamers from sequenced random regions^30^. All generated sequences in the next-generation sequencing (NGS) pool were grouped based on their shared sequence features. A representative sequence from each cluster was selected for further analysis to identify the most prevalent aptamer sequences. This approach allowed us to group putative FMDV aptamer sequences and identify the most prominent sequence for further characterization.

Structural analysis of FMDV Apt and molecular modeling with FMDV VP1 highlighted key features used to select structures and models. Hydrogen bond-based intramolecular base pairing is one feature used to select higher-order structures of the identified aptamer sequences as determined by structural modeling^31,32^. A second feature used to identify aptamer structures is the deoxyribose-phosphodiester backbone that can fit into six different torsion angles, thus providing diversified secondary and tertiary structure formations^15,33^. Molecular docking using HDOCK has enabled us to rank these models based on docking and confidence scores, as well as ligand RMSD values^19,34^.

Minor adjustments in how a ligand or ligand–receptor assembly (including aptamers) is positioned or rotated can produce substantial shifts in the calculated ligand RMSD, even when the true binding configuration or biological significance remains essentially the same. Although ligand RMSD alone is not a perfect indicator for evaluating every docking result, it is still commonly employed to assess the conformational stability of large biomolecules during molecular dynamics simulations. Altogether, the molecular docking simulation and analysis provide a more complete picture of the interactions between FMDV Apt and FMDV VP1.

This study supports the notion that the generated FMDV VP1 aptamer, FMDV Apt, can be incorporated into an ALISA-based microplate platform to detect FMDV VP1 with robust binding affinity and selectivity. This method did not elicit cross-reactivity with non-target samples, suggesting stable and exclusive interaction, and highlights its potential for integration with other detection platforms. As expected, a linear relationship and no cross-reactivity were observed during the assay, since SVA VP1 was also used as the negative selection protein bait in the SELEX procedure. This strategy has provided excellent specificity and exclusivity of FMDV Apt as the capture and detection element when used in detection platforms, especially against SVA.

The newly developed aptamer-based screening method would facilitate efficient and inexpensive testing and surveillance of potential biohazard risks presented by deadly viral agents. This aptamer-based approach supports potential application in FMDV screening in addition to traditional immune-based technologies, as current diagnosis relies on serologic and immuno-based methods that commonly encounter cross-reactivity, cost issues, and require highly trained staff.

Future efforts will focus on enhancing the sensitivity of the assay and further validating specificity by testing additional non-target agents that cause clinical symptoms similar to those of FMDV infection, as well as related environmental samples. In addition, performing more comprehensive replicative experiments to assess the consistency and robustness of ALISA – from structural predictions to side-by-side comparison with ELISA (direct, indirect, and sandwich) and other available commercial kits for detecting FMDV antigens. Similarly, using an FMD pseudovirus in this platform will further validate our findings. In addition, various clinical and environmental matrices will be included during testing to challenge the assay’s robustness in samples with high background noise and interferents.

Overall, this study provides strong evidence for using a DNA-based aptamer to detect VP1 of FMDV serotype O and contributes to the development of an aptamer-based diagnostic platform.

## Materials and methods

### Systematic evolution of ligands by exponential enrichment (SELEX)

Systematic Evolution of Ligands by Exponential Enrichment (SELEX) was performed as previously reported^28,35^, with minor modifications described below. FMDV viral protein 1 (VP1) is an exposed surface protein that acts as a binding target in detecting FMDV. Briefly, His-tagged FMDV serotype O VP1 recombinant protein (0.1 nmol) (FMDOVP15-R-10, Alpha Diagnostic International) in binding buffer (2.5 mM MgCl_2_, 1 mM Heparin sodium salt, 3 nmol Sheared Salmon sperm DNA (ThermoFisher), 0.02% Tween-20, and 1× PBS (pH 7.4)) was immobilized on Dynabeads His-tag Isolation and Pulldown beads (ThermoFisher). Next, A library of ssDNA sequences (100 µM) with fixed 5’ and 3’ regions and a central 45 nucleotide random stretch (Table 1) was incubated for 1 h with the bead-FMDV VP1 complex then washed using washing buffer (2.5 mM MgCl_2_, 0.02% Tween-20, 1× PBS) to remove ssDNA that did not bind to the complex. The bound ssDNA was then used as a template for a preparative PCR step using primers (FMDV Aptamer Fwd and Rev) (0.5 µM) that amplify the fixed 5’ and 3’ regions of the ssDNA library sequences (Table 1) (IDT) and Phusion Flash High-Fidelity PCR Master Mix (1×) (ThermoFisher) where the optimal cycle number for a subsequent amplification PCR was determined (Supplementary Figure S1 and Table S1). This preparative PCR product was then used for the amplification PCR step and sub-library generation. The amplified PCR products were pooled and reduced using a CentriVap micro-IR vacuum concentrator (Labconco) at 3,000 x g and 56 °C prior to denaturation using a 10% Mini-PROTEAN Tris-Borate-EDTA (TBE) Urea gel (Biorad). The FMDV Aptamer Rev primer contains a 5’ 20 nucleotide polyT tail (Table 1) that lengthens the ssDNA antisense strand and distinguishes it from its partner to allow separation and selection of these bands. These separated ssDNA bands were viewed using a blue/white light transilluminator (ThermoFisher), and the target sense band was excised. Electro-elution of the excised bands was performed using a 3.5 kDa cut-off dialysis tube at 120 V for 20 min, followed by ethanol precipitation to recover the ssDNA^28^. This procedure was repeated up to 8 rounds. A non-target protein, Senecavirus A (SVA) VP1 (0.1 nmol), was introduced in round 5 for negative selection of nonspecific DNA. In subsequent rounds, the amount of FMDV VP1 was gradually reduced (0.1 nmol to 0.001 nmol) and the incubation time with the ssDNA pool was shortened from 1 h to 10 min, while the wash time and amount of random sperm ssDNA were increased. The final products were amplified using extended primers (NGS Ext Fwd and Rev) (Table 1) (IDT) and used for next-generation sequencing (NGS) (Azenta Life Sciences) and data processing.

**Table 1:** Sequences of SsDNA library, conventional PCR primers, and aptamers used and generated in the study.

Name	Sequence
ssDNA Library	5’ – ATCCAGAGTGACGCAGCA – 45 N – TGGACACGGTGGCTTAGT − 3’
FMDV Aptamer – Fwd	5’ – AATTGCCCCCAGAGTGGATG – 3’
FMDV Aptamer – Rev	5’ – TTTTTTTTTTTTTTTTTTTTGTATACGATGACGCCGGGAG – 3’
NGS Ext – Fwd	5’ – CGTCTA ATTGTAACGGGTGACTGTATAGCTAATAAT CCGAATCCAGAGTGACGCAGCA – 3’
NGS Ext – Rev	5’ – GCAGAATAGTTACGTGACATGTCTTGCCTGAAGTCAGAT CACTAAGCCACCGTGTCCA – 3’
FMDV Apt	5’ – ATCCAGAGTGACGCAGCAAGAGACCTGCAAGG CAAGCGATTTAAGTGGCACCCCCAGGGACCATGGACACG GTGGCTTAGT – 3’
FMDV Apt Flour	5’ – 6-FAM/ATCCAGAGTGACGCAGCAAGAGACCTGCAAGG CAAGCGATTTAAGTGGCACCCCCAGGGACCATGGACACG GTGGCTTAGT – 3’
FMDV Apt Biotin	5’ – Bio/ATCCAGAGTGACGCAGCAAGAGACCTGCAAGG CAAGCGATTTAAGTGGCACCCCCAGGGACCATGGACACG GTGGCTTAGT – 3’

### Selection and characterization of aptamers

Processing of the NGS data was performed as previously described^28^. Briefly, Python (version 3.12) was used to write a frequency or sequence script to cluster and rank sequences in the aptamer pool. Next, the identified sequences were subjected to a simulation pipeline to generate predicted secondary and tertiary DNA structures and simulate DNA docking to the FMDV VP1 target protein. DNA secondary structure predictions were generated by RNAsoft CombFold^16^ and visualized using RiboSketch^17^. DNA tertiary structures were generated using Discovery Studio Visualizer (Biovia) and CHARMM-GUI^18^. Molecular docking simulations of FMDV Apt and VP1 were conducted using HDOCK^19^. Initially, VP1 structure (pdb file) was downloaded from www.rcsb.org and used as a receptor molecule in HDOCK^19^ which generated docking models based on docking scores^19^. Lastly, the HDOCK generated FMDV VP1-aptamer complexes were analyzed using PLIP (version 2.3.0) to identify and analyze interactions between FMDV VP1 and aptamer sequences. Fluorescently labeled FMDV Apt was tested to measure interactions with molecular targets using a previously reported magnetic bead-based isocratic elution method^24^ with minor modifications. In brief, the FMDV VP1 recombinant protein in binding buffer was immobilized onto His-tag Isolation and Pulldown Beads. Next, a π-π stacking range of fluorescently labeled FMDV Apt (10 nM – 1,000 nM) with no linear correlation below 1 ng/mL in binding buffer was heated at 90 °C for 5 min, cooled on ice for 15 min, and 90 µL of this solution was incubated with 90 µL of VP1 recombinant protein derivatized magnetic beads at 37 °C for 1 h. The supernatant was discarded, and the beads were washed 4 times with 60 µL of wash buffer for 2 min each to remove any unbound aptamer. The bound aptamers were then eluted from the complex with 180 µL of TE buffer (10 mM Tris HCl and 1 mM EDTA, pH 8) and incubated at 90 °C for 10 min. This solution was placed on a magnet for 2 min to collect the supernatant containing the aptamers. A second elution step was performed to ensure complete recovery of aptamers, and the elutions were pooled. The fluorescence emission for each aptamer concentration was measured (excitation λ = 490 nm; emission λ = 520 nm) using a SpectraMax M2e Multi-Mode Microplate Reader (Molecular Devices). The average fluorescence intensity was plotted as a function of aptamer concentration and used to calculate the response using Eq. (1), as previously reported^28^, where *A* is the absorbance intensity at a given concentration of FMDV VP1, and $A_\theta$ is the absorbance intensity in the absence of FMDV VP1. *K*_d_ was calculated using OriginLab (OriginLab Corporation) software. Briefly, the calculated responses from Eq. (1) were analyzed to generate the mean ± S.D. using the Descriptive Statistics option of OriginLab. Next, the aptamer concentration was plotted against the average mean, and a nonlinear fitting option was used. The *K*_d_ function was then used to calculate *K*_d_ values (nM). The experiment was performed three times in triplicate (*n* = 3).

1 $$\:Response=\frac{A-A_\theta}{A}$$

### Aptamer-linked immobilized sorbent assay (ALISA)

The application of FMDV Apt in an ALISA platform was adapted from a previous report^23^ with modifications described below. 100 µL of FMDV Apt (1 µM) was added to each well of a 96-well medium binding microplate and incubated at room temperature for 2 h. 100 µL of SuperBlock Blocking Buffer (ThermoFisher) was then added atop the aptamer solution and allowed to stand at room temperature for 30 min. After this, the well contents were discarded, the wells were washed with blocking buffer, and the plate was air-dried for 10 min. Next, BSA (1%) was added to each well and the plate was incubated for 30 min at room temperature. After discarding the well contents, 100 µL of various concentrations (0.05 ng/mL – 2000 ng/mL) of recombinant FMDV serotype O VP1, Seneca Valley virus VP1 (SVA VP1, SEVC15-R-10) and H1N1 hemagglutinin (HA) (H1N1-03-R-10) (Alpha Diagnostic International) proteins in binding buffer were added and incubated at room temperature for 1 h with gentle shaking. This solution was discarded, and the wells were washed twice with 100 µL of PBST (1× PBS containing 0.1% Tween-20). Next, 100 µL of biotinylated FMDV Apt (1 µM) (Table 1) solution was added to individual wells and incubated at room temperature for 1 h. The wells were washed twice with 100 µL of PBST followed by the addition of 100 µL of streptavidin-horseradish peroxidase (HRP) conjugate (Millipore Sigma) and incubated for 30 min at room temperature with shaking. The microplate was then washed twice with 100 µL of PBST. Next, 100 µL of 3,3′,5,5′-tetramethylbenzidine (TMB) One Solution (Promega) was added to each well, and the plate was kept in the dark at room temperature for approximately 10 min until the solution turned blue. The reaction was next quenched with 100 µL of Stop Solution for TMB Substrates (ThermoFisher), and the A_450 nm_ was measured using a Biotek Microplate Reader (Agilent).

### Statistical analysis

Microsoft Excel program was used for linear regression analysis which generated the standard error and calibration curve with estimated slope. Both standard error and slope were used to calculate the LOD = (3.3 × σ) /*S* and LOQ = (10 × σ) /*S*), where: σ = the S.D. of the response; *S* = the slope of the calibration curve as defined by the International Conference on Harmonization (ICH) in analytical method validation, specifically in the ICH Q2(R2) guideline. All samples were tested in triplicates, and the mean of the data ± S.D. was analyzed using JMP Pro 16 software and Microsoft Excel program by one-way ANOVA with Fisher’s least significant difference (LSD) post hoc analysis to confirm the significant differences between groups (*p* < 0.05).

## Supplementary Information

Below is the link to the electronic supplementary material.

Supplementary Material 1

## Data Availability

All datasets generated for this study are included in the manuscript and/or the Supplementary Materials. Sets of frequency or sequence scripts in Python version 3.12. to analyze NGS data by clustering and ranking aptamer sequences can be found in [https://github.com/Wu-Microbiology/aptamerdocking](https:/github.com/Wu-Microbiology/aptamerdocking) . For additional relevant data, please contact the corresponding author.
