# Supplementary material for: Aptamer-linked immobilized sorbent assay for detection of VP1 of foot and mouth disease virus serotype O
Source: Sci Rep. 2026 Feb 3;16:5453. doi: 10.1038/s41598-025-34793-8 (PMC12886914; doi:10.1038/s41598-025-34793-8)
Supplement: Supplementary file 1 — Supplementary Material 1 [file 41598_2025_34793_MOESM1_ESM.pdf]

Supplementary Material

Aptamer-Linked Immobilized Sorbent Assay for Detecting Foot and Mouth Disease Virus VP1 Serotype O

Irwin A. Quintela[1], Raymondo Lopez-Magaña[1], Anya Hwang[1], Tyler Vasse[1], and Vivian C.H. Wu[1*]

[1] Produce Safety and Microbiology Research Unit, U.S. Department of Agriculture, Agricultural Research Service, Western Regional Research Center, Albany, CA 94710, USA

*** Correspondence:**
Corresponding Author
vivian.wu@usda.gov

(a)

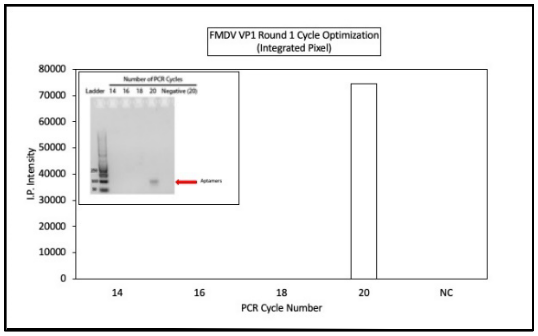
FMDV VP1 Round 1 Cycle Optimization
(Integrated Pixel)
I.P. Intensity
PCR Cycle Number
Number of PCR Cycles
Ladder 14 16 18 20 Negative (20)
Aptamers
80000
70000
60000
50000
40000
30000
20000
10000
0
14
16
18
20
NC

(b)

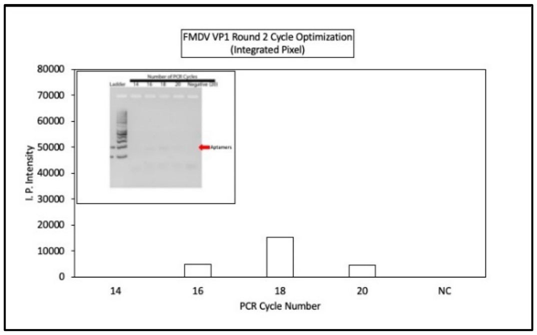
FMDV VP1 Round 2 Cycle Optimization
(Integrated Pixel)
I. P. Intensity
PCR Cycle Number
80000
70000
60000
50000
40000
30000
20000
10000
0
14
16
18
20
NC

(c)

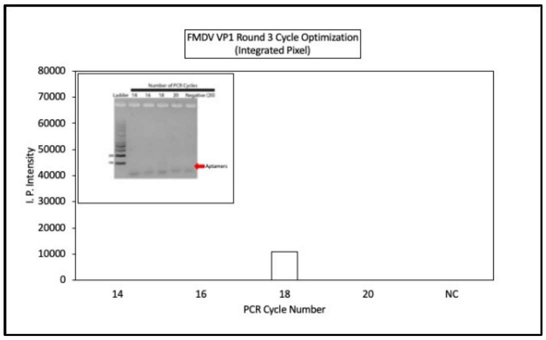
FMDV VP1 Round 3 Cycle Optimization
(Integrated Pixel)
I. P. Intensity
PCR Cycle Number
80000
70000
60000
50000
40000
30000
20000
10000
0
14
16
18
20
NC

(d)

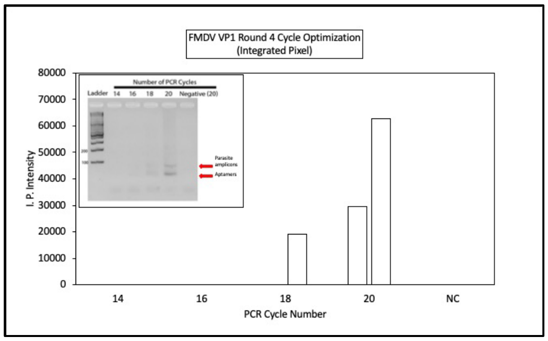
FMDV VP1 Round 4 Cycle Optimization
(Integrated Pixel)
I. P. Intensity
PCR Cycle Number
Number of PCR Cycles
Ladder 14 16 18 20 Negative (20)
Parasite amplicons
Aptamers
80000
70000
60000
50000
40000
30000
20000
10000
0
14
16
18
20
NC

(e)

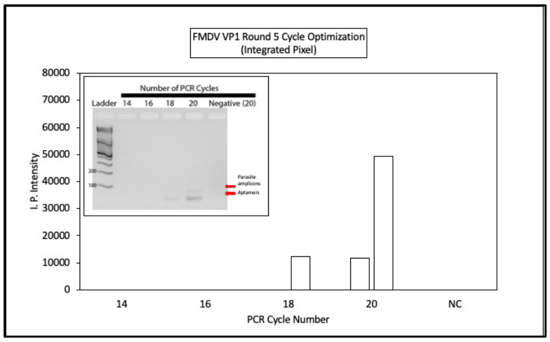
FMDV VP1 Round 5 Cycle Optimization
(Integrated Pixel)
I. P. Intensity
PCR Cycle Number
Number of PCR Cycles
Ladder 14 16 18 20 Negative (20)
200
100
Parasite amplicons
Aptamers
80000
70000
60000
50000
40000
30000
20000
10000
0
14
16
18
20
NC

(f)

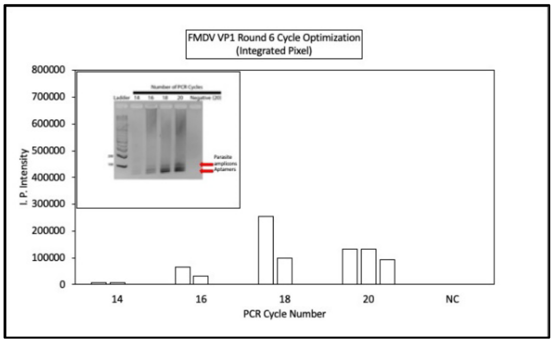
FMDV VP1 Round 6 Cycle Optimization
(Integrated Pixel)
I. P. Intensity
PCR Cycle Number
800000
700000
600000
500000
400000
300000
200000
100000
0
14
16
18
20
NC

(g)

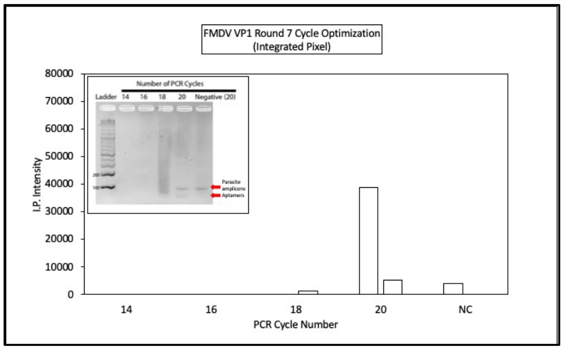

Supplementary Figure S1. Agarose gels with the corresponding bar graphs showing Integrated Pixel (I.P.) during preparative conventional PCR from SELEX Rounds 1 – 7. (a) Round 1, (b) Round 2, (c) Round 3, (d) Round 4, (e) Round 5, (f) Round 6, and (g) Round 7. The optimal PCR cycle number is the one that yields the greatest peak intensity, typically ranging from 18 to 20 cycles.

Supplementary Table S1. SELEX PCR conditions. Conditions used for amplification in PCR. The optimal PCR cycles were identified during the preparative PCR step.

Step	Temperature (°C)	Time (s)
Denaturation	95	120
Amplification (18-20 cycles)		
Denaturation	95	30
Annealing	56	30
Extension	72	30
Final Extension	72	5
Hold	4	

Supplementary Table S2. Summary of the top eight molecular docking models using HDOCK (Yan et al., 2017). Molecular models were ranked based on individual docking scores.

Rank/Model	Docking Score	Confidence Score	Ligand RMSD (Å)
Model 1	-301.3	0.9535	509.29
Model 2	-288.30	0.9408	477.88
Model 3	-283.13	0.93448	521.94
Model 4	-273.33	0.9218	540.65
Model 5	-270.90	0.9182	540.65
Model 6	-263.76	0.9068	491.93
Model 7	-263.67	0.9066	473.65
Model 8	-263.07	0.9056	518.16

Supplementary Table S3(a). Analysis of the absorbance ($A_{450\ nm}$) response graph of FMDV Apt with various concentrations of FMDV VP1 (ng/mL).

**Concentration of FMDV VP1 (ng/mL)**	**$A_{450\ nm}$ ± Std. Deviation**
0	0.984090535 ± 0.1405953
0.50	0.984807113 ± 0.050251
1.0	1.084561907 ± 0.0216463
2.5	1.22275704 ± 0.0534454
5.0	1.294955573 ± 0.0757962

Supplementary Table S3(b). Regression statistics.

Regression Statistics	
Multiple R	0.964835
R Square	0.930906
Adjusted R Square	0.907874
Standard Error	0.042657
Observations	5

Supplementary Table S3(c). One-way ANOVA analysis.

	df	*SS*	*MS*	*F*	*Significance F*
Regression	1	0.073547714	0.073548	40.419	0.007874136
Residual	3	0.005458897	0.00182		
Total	4	0.079006611			

Supplementary Table S3(d). **LOD and LOQ**. LOD was calculated as 3.3 x Std. error divided by the slope. LOQ was calculated as 10 x Std. error divided by the slope.

	Coefficients	*Standard Error*	*t Stat*	*P-value*	*Lower 95%*	*Upper 95%*	*Lower 95.0%*	*Upper 95.0%*
Intercept	1.003794	0.025801071	38.90511	3.74E-05	0.921683018	1.085904062	0.921683	1.085904
X Variable 1	0.064585	0.010158768	6.357594	0.007874	0.032255584	0.096915051	0.032256	0.096915

LOD = (3.3 x 0.025801071) / 0.064585 = **1.31 ng/mL**

LOQ = (~~3.3~~ x 0.025801071) / 0.064585 = **3.99 or 4 ng/mL**

References

Yan, Y., Zhang, D., Zhou, P., Li, B., and Huang, S.-Y. (2017). HDOCK: a web server for protein–protein and protein–DNA/RNA docking based on a hybrid strategy. *Nucleic acids research* 45**,** W365-W373.
